# Dynamic Characteristics of Additive Manufacturing Based on Dual Materials of Heterogeneity

**DOI:** 10.3390/polym17131793

**Published:** 2025-06-27

**Authors:** Hsien-Hsiu Hung, Shih-Han Chang, Yu-Hsi Huang

**Affiliations:** 1 Department of Mechanical Engineering, National Taiwan University, Taipei 10617, Taiwan; d06522015@ntu.edu.tw; 2 Department of Mechanical Engineering, National Taiwan University of Science and Technology, Taipei 10607, Taiwan

**Keywords:** additive manufacturing, viscoelastic material, composite material, natural frequency, Prony series

## Abstract

This study aims to establish a methodology that integrates experimental measurements with finite element analysis (FEA) to investigate the mechanical behavior and dynamic characteristics of soft–hard laminated composites fabricated via additive manufacturing (AM) under dynamic excitation. A hybrid AM technique was employed, using the PolyJet process based on stereolithography (SLA) to fabricate composite beam structures composed of alternating soft and hard materials. Initially, impact tests using a steel ball on cantilever beams made of hard material were conducted to inversely calculate the first natural frequency via time–frequency analysis, thereby identifying Young’s modulus and Poisson’s ratio. For the viscoelastic soft material, tensile and stress relaxation tests were performed to construct a Generalized Maxwell Model, from which the Prony series parameters were derived. Subsequently, symmetric and asymmetric multilayer composite beams were fabricated and subjected to impact testing. The experimental results were compared with FEA simulations to evaluate the accuracy and validity of the identified material parameters of different structural configurations under vibration modes. The research focuses on the time- and frequency-dependent stiffness response of the composite by hard and soft materials and integrating this behavior into structural dynamic simulations. The specific objectives of the study include (1) establishing the Prony series parameters for the soft material integrated with hard material and implementing them in the FE model, (2) validating the accuracy of resonant frequencies and dynamic responses through combined experimental and simulation, (3) analyzing the influence of composite material symmetry and thickness ratio on dynamic modals, and (4) comparing simulation results with experimental measurements to assess the reliability and accuracy of the proposed modeling framework.

## 1. Introduction

From the perspective of composite material structures, materials composed of two or more constituents with distinct properties can achieve complementary integration of individual material advantages, resulting in synergistic effects. This enhances the overall performance of composites beyond what can be achieved by any single material, thereby meeting diverse application demands. Consequently, composite materials are widely utilized in industries such as aerospace, architecture, and automotive engineering. With stereolithographic additive manufacturing technologies, heterogeneous laminated structures composed of multiple materials can be fabricated without the need for additional adhesives, enabling direct integration between layers. In this study, conventional material testing methods are integrated with finite element analysis for modifying material constants. Through experimental validation of dynamic behavior, the influence of various layered configurations of soft and rigid materials on structural stiffness and modal responses is systematically analyzed.

In 2002, Li et al. [1] extensively utilized the Fused Deposition Modeling (FDM) printing technique to conduct research. Their study first explored the relationship between fiber spacing and density. Four different spacing configurations were subjected to tensile tests to determine Young’s modulus, Poisson’s ratio, and shear modulus. In 2007, Lee et al. [2] conducted a comparative study using Fused Deposition Modeling (FDM), Stereolithography (SLA), and powder-based binder jetting to fabricate cylindrical specimens for compression testing in accordance with ASTM D695. The materials used were acrylonitrile butadiene styrene (ABS) for FDM, a resin mixed with nanoparticles for SLA, and gypsum for the powder binder jetting process. With advancements in manufacturing processes, research has expanded beyond single-material properties to developing composite materials, that is, dual-material or above systems. In recent years, both 3D-printed and metallic materials have reached a high level of maturity. In this study, the combination of dual materials includes not only rigid materials but also polymeric materials. In this study, since soft and hard materials need to be combined into bulk within a single manufacturing process, FDM manufacturing leads to delamination. Therefore, we used SLA additive manufacturing to print the mixed hard–soft multilayered structure for the research.

Most polymeric materials exhibit viscoelastic behavior, particularly under prolonged exposure to time-dependent loading and high-temperature processing environments, leading to pronounced viscoelastic characteristics. Extensive research has been conducted on viscoelastic modeling, with well-established linear viscoelastic models effectively describing the mechanical behavior of materials as they respond to changes in time and temperature. In 1980, Ferry [3] established that the modulus of viscoelastic materials is intrinsically related to both temperature and time. This relationship can be described using the time–temperature superposition principle. The modulus curves obtained at different temperatures can be shifted along the time axis relative to a reference temperature, thereby enabling the construction of a master curve that describes the material’s modulus as a function of time under varying temperature conditions. However, the study does not consider temperature effects, and all experimental analyses are conducted at a constant ambient temperature of 25 °C. Most viscoelastic materials exhibit a Poisson’s ratio in the range of 0.45 to 0.49. In 2008, Mott et al. [4] pointed out that when the Poisson’s ratio of a viscoelastic material approaches 0.5, it is often assumed that the bulk modulus tends to infinity. Their experimental measurements indicated that the bulk modulus should be considered negligible for viscoelastic materials approaching an incompressible state. Consequently, in simulation analyses, such materials are often defined as incompressible. In 2010, Der-Song et al. [5] utilized finite element analysis (FEA) to simulate the effects of temperature-induced warpage in polydimethylsiloxane (PDMS) when applied in medical sealing applications. Additionally, they experimentally investigated how the material’s strength varied with frequency. Their study provided valuable insights into the key considerations for setting material properties in viscoelastic modeling and simulation. Subsequently, extensive experimentation is required to obtain the necessary parameters for theoretical and analytical modeling of viscoelastic materials. In 2014, Bang and Jeong [6] utilized a rheometer and stress relaxation mode to measure the shear storage modulus, shear loss modulus, and shear relaxation modulus of polyurethane (a polymer material). They further investigated and compared various analytical models through curve fitting to evaluate their accuracy and applicability. This study also employed the aforementioned material testing methods. However, based on the combination of soft and hard materials, it examines the applicability of material constants for analysis through the dynamic impact characteristics.

This approach has been applied to the beam-type specimens made of viscoelastic materials fabricated through additive manufacturing in this study. Li [7] proposed a method for simultaneously analyzing the static and dynamic behavior of functionally graded beams (FGBs) and derived a fourth-order partial differential equation. By simplifying it into the Timoshenko, Rayleigh, and Euler–Bernoulli beam equations, the study identified the phenomenon of dual weight ratios in FGB, which holds significant value for engineering structural analysis. Ma and Huang [8] conducted a study validating the applicability of PVDF sensors in dynamic strain measurements, particularly highlighting their advantages in low-strain and high-frequency vibration environments. By comparing the measurements obtained from PVDF sensors with finite element method (FEM) and theoretical calculations, the study confirmed the accuracy of the sensor’s measurements. Chuang and Li [9] developed a fiber Bragg grating (FBG) displacement and strain sensing system to experimentally investigate the dynamic behavior of a cantilever beam under impact loading or moving mass loading. The study includes using FEM to validate the measurement accuracy of the FBG displacement sensor for transient response and to analyze different vibration modes of the cantilever beam, including bending mode, transverse bending mode, and torsional mode. This study references the same theoretical approach and FBG sensing system to examine the relationship between impact or moving mass-induced transient responses and vibration modes by laser Doppler vibrometer.

The dynamic response of viscoelastic composite materials is influenced by both material composition and structural characteristics, making them suitable for applications in aerospace, mechanical vibration control, and acoustic material design. This work provides the mechanical framework for this study, encompassing fundamental theories, strength analysis, and dynamic behavior. Gibson [10] integrated classical and modern composite material mechanics theories, covering material selection, mechanical behavior, strength analysis, and dynamic behavior. Campoli et al. [11] investigated the mechanical property prediction methods for open-cell metallic biomaterials fabricated through additive manufacturing. The study proposed a novel approach based on FEM to predict the mechanical behavior of these materials. This research references this effective numerical method to predict the mechanical behavior of additively manufactured materials. Yavari et al. [12] investigated the influence of bio-functionalizing surface treatments on high-porosity titanium alloy structures’ static and fatigue performance. These structures exhibit similarities to additively manufactured materials. This study references the mechanical performance testing methods presented in the literature for evaluating similar material characteristics. Lin et al. [13] studied the mechanical properties of polydimethylsiloxane, a polymer material widely used in biomedical microelectromechanical systems (Bio-MEMS). The primary objective was to develop a more accurate viscoelastic constitutive model for cellular traction force measurement, as most previous studies have treated PDMS as a linear elastic material, potentially leading to measurement errors. This study references the literature and establishes a viscoelastic constitutive equation based on the generalized Maxwell model, which is used for bonding multilayered, additively manufactured materials with different hardness levels. Additionally, the Prony series is employed to fit the generalized Maxwell model, and various testing methods are utilized to validate the material parameters and resonance frequencies of the additively manufactured materials. This systematic review by Nazir et al. [14] examines the design principles, material selection strategies, and challenges of multi-material additive manufacturing, offering insights into hard–soft composite structures. The study employs experimental design and FEM, including impact testing and inverse material constant identification, to establish theoretical and empirical references. The reviewed modeling approaches provide valuable guidance for refining Prony-based and Maxwell modular strategies. Glaesener et al. [15] integrate the generalized Maxwell viscoelastic model with FEM to simulate the time-dependent behavior of polymer-based 3D lattice metamaterials. Using corotational beam elements, the study effectively captures microscale viscoelasticity while enabling macroscale structural simulations, particularly relevant for impact absorption and vibration damping. Their parameter fitting and validation methods, especially for Young’s modulus, Poisson’s ratio, and Prony coefficients, are useful. Ferreira [16] introduces an image-based numerical homogenization method utilizing the Maxwell model and Prony series to characterize viscoelastic materials for dynamic composite system analysis. This approach supports FEM simulations for SLA-printed structures.

Bozkurt [17] developed a data-driven computational framework for homogenous viscoelastic porous elastomers, enhancing predictive analysis of multilayer composites. Salahshoor and Ortiz [18] proposed a novel algorithm transforming viscoelastic experimental data into frequency-domain responses without relying on traditional modeling assumptions. Their method quantifies stress–strain historical pairs with high accuracy, providing insights into dynamic modal analysis of hybrid composite beams. Marino et al. [19] designed EUCLID, a frequency-domain identification framework employing sparse regression (Lasso) and machine learning clustering (k-means). This efficient approach extracts compact material models, mitigating ill-posedness issues. Combined with digital image correlation (DIC), their method proves valuable for structural material parameter inversion. Batt et al. [20] analyzed a linearized viscoelastic rod under free and forced vibrations using the Maxwell–Weichert model and Prony series. Their stress relaxation testing methodology aids in the validation of modal frequencies and damping effects. Duan et al. [21] introduced a cohesive zone model (CZM) for rate-dependent viscoelastic interfaces in sensor systems, establishing a robust framework validated via finite element simulations. Estermann et al. [22] developed a viscoelastic Mori–Tanaka micromechanical model (vMTM) for biological soft tissue simulation. Their approach predicts effective moduli and energy dissipation behavior using stress relaxation data, supporting biomimetic tissue modeling applications.

Wallace et al. [23] proposed an efficient contact modeling framework for multilayered viscoelastic materials, employing the Maxwell model and Prony series parameters. Their semi-analytical method, coupled with FFT algorithms, enhances simulation efficiency and informs laminated structure modeling. Zobeiry et al. [24] advocated for differential-form constitutive modeling over traditional integral approaches, ensuring numerical stability and scalability. Their workflow effectively integrates experimentally derived Prony parameters into FEM. Abayazid and Ghajari [25] investigated PolyJet-fabricated elastomers Agilus30 and Tango, developing a visco-hyperelastic model integrating an Ogden formulation with five Prony series terms. The study revealed that print orientation, particularly along the Z-axis, significantly influences mechanical strength, highlighting the need to carefully consider build direction when designing flexible structures. The modeling approach and analytical framework proposed in their study provide a valuable reference for the development of elastomeric material models and FEM in this work.

## 2. Basic Theory

This study applied Hooke’s Law, Euler–Bernoulli beam theory, and the generalized Maxwell model, which are classical frameworks for the SLA-printed heterogeneous dual-material structures (hard–soft laminates). Through cantilever beam impact tests, we inversely calculated equivalent Young’s modulus and Poisson’s ratio, demonstrating the significant influence of layer thickness and sequence on modal frequencies and damping characteristics.

### 2.1. Hooke’s Law Stress–Strain Relationship

In solid mechanics, Hooke’s Law serves as a fundamental theory for describing the elastic deformation behavior of materials. For homogeneous, isotropic, and prismatic materials, whose mechanical properties are identical, the linear elastic response under loading can be characterized using a single material constant—Young’s modulus *E*. When a material is subjected to axial stress, and the strain remains within the elastic limit, a linear relationship exists between the stress *σ* and strain *ε*. This relationship can be expressed as

(1) σ=E×ε

Equation (1) characterizes a resistance of material to deformation. This formulation captures the essence of elastic deformation in isotropic solids under uniaxial loading, assuming small strain stability under linear elasticity conditions. Hooke’s Law is applicable only within the linear elastic regime, where deformations are fully recoverable upon unloading and no permanent strain remains. Once external forces exceed this range, the material may exhibit plastic deformation or failure. Thus, Hooke’s Law is particularly suited for predicting the stress–strain behavior of structural elements such as beams and rods during their elastic phase of loading. A tiny steel ball was dropped from minimal heights onto a cantilever beam to induce vibrations within the elastic range for experimental conditions in the paper.

### 2.2. Viscoelasticity Theory

**Figure 1:**
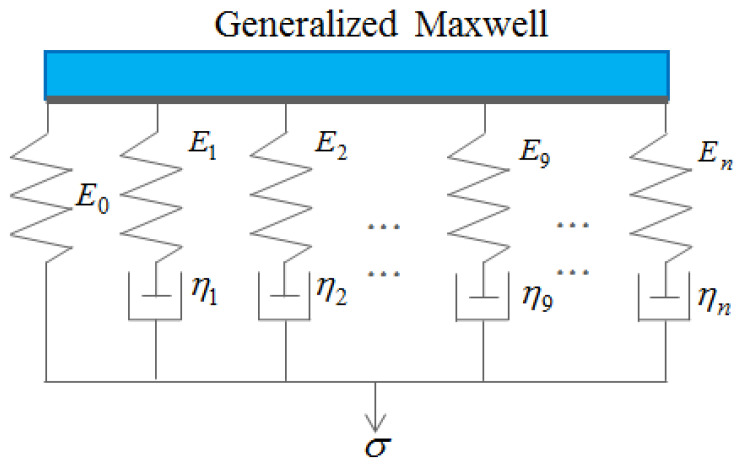
Generalized Maxwell model.

The mathematical model employed in this study is the generalized Maxwell model for viscoelastic material, which provides a more accurate representation of real material behavior. The mathematical formulation of this model is expressed using the Prony series, which can be represented as follows:

(2) $$g_{R}(t)=1-\sum_{i=1}^{N}g_{i}\left[1-\exp^{\frac{-t}{\tau_{i}}}\right]=\frac{E(t)}{E_{0}}$$

(3) $$k_{R}(t)=1-\sum_{i=1}^{N}k_{i}\left[1-\exp^{\frac{-t}{\tau_{i}}}\right]=\frac{K(t)}{K_{0}}$$

where *E*_0_ represents the initial elastic modulus, while *E*(*t*) denotes the time-dependent relaxation modulus characterizing the variation of elastic response over time. Similarly, *K*_0_ is defined as the initial bulk modulus, and *K*(*t*) corresponds to the time-dependent bulk modulus. The parameter *τ_i_* refers to the characteristic relaxation time associated with each term in the Prony series, and *N* denotes the total number of terms used in the Prony series representation. This mathematical formulation enables accurate modeling of viscoelastic behavior under time-dependent loading conditions, as shown in Figure 1.

### 2.3. Cantilever Beam Vibration

This section introduces the Bernoulli–Euler beam theory, deriving the governing equations for bending, lateral, torsional, and axial modes from the fundamental governing equations.

#### 2.3.1. Bending Modes and Lateral Modes of a Cantilever Beam

**Figure 2:**
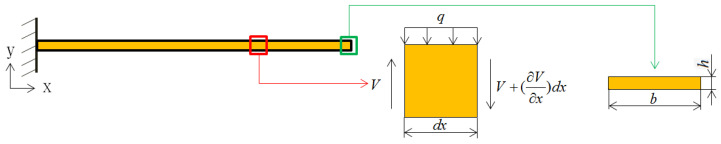
A small region of a cantilever beam undergoing bending deformation.

According to the assumptions of the Euler–Bernoulli beam theory, the deformation caused by shear can be neglected. In other words, after the beam undergoes bending under loading, the cross-sections initially perpendicular to the neutral axis remain planar and perpendicular to the neutral axis, as illustrated in Figure 2. The equilibrium governing this deformation is described by the following equation.

(4) $$\frac{\partial^{2}y}{\partial x^{2}}=-\frac{M}{EI},\;I=\frac{bh^{3}}{12}$$

(5) $$\left(V+\frac{\partial V}{\partial x}dx\right)-V+qdx=\rho Adx\frac{\partial^{2}y}{\partial t^{2}}$$

where the moment of inertia is *I*, in which the rectangular section is considered in the experimental measurement with width *b* and height *h*. The *ρ* and *A* are the density and area, respectively. By incorporating the relationship between shear force, *V*, and bending moment, *M*, from the mechanics of materials into Equation (5), the governing equation can be derived.

(6) $$\frac{\partial^{2}}{\partial x^{2}}\left(EI\frac{\partial^{2}y}{\partial x^{2}}\right)+\rho A\frac{\partial^{2}y}{\partial t^{2}}=q(x,t)$$

After using the separation of variables method to calculate, substitute the boundary conditions into

(7) $$Y(0)=Y^{\prime }(0)=Y^{\prime \prime }(l)=Y^{\prime \prime \prime }(l)=0$$

Finally, the characteristic equation is

(8) cosβlcoshβl=−1

The first six solutions are

$$\beta_{1}l=1.875,\;\beta_{2}l=4.694,\;\beta_{3}l=7.855,\;\beta_{4}l=10.996,\;\beta_{5}l=14.137,\;\beta_{6}l=17.279$$

where

$$\;\beta_{i}^{4}=\frac{\omega_{i}^{2}m}{EI}$$

This study utilized the first eigenvalue to inversely calculate the Young’s modulus. The Young’s modulus, with other material constants, was then input into theoretical calculations and FEM simulations to obtain comparisons of several resonant frequencies. This approach was used to verify the accuracy of the experimentally determined material constants for simulation purposes.

#### 2.3.2. Torsion Mode of Cantilever Beam

**Figure 3:**
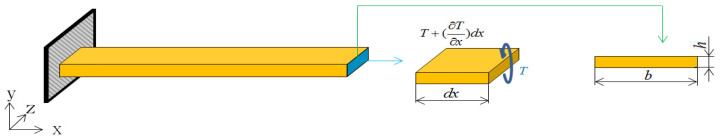
Torsional behavior of an infinitesimal segment in a cantilever beam.

The following figure shows a free-body diagram of a small segment of a cantilever beam with length *dx*. Based on this infinitesimal element, as shown in Figure 3, the torsional equilibrium equation can be expressed as follows:

(9) $$-T+\left(T+\frac{\partial T}{\partial x}dx\right)=\rho Jdx\frac{\partial^{2}\theta }{\partial t^{2}}$$

Let the diameter function be

(10) $$\theta (x,t)=\Theta (x)e^{iwt}$$

The general solution can be written as

(11) $$\Theta (x)=C_{1}\sin k_{n}x+C_{2}\cos k_{n}x,\;k_{n}=\frac{w_{n}}{c_{S}}$$

Then, according to the boundary condition of the torsional of the cantilever beam,

(12) $${\left.\theta (x,t)\right|}_{x=0}=0\;C_{T}{\left.\frac{\partial \theta (x,t)}{\partial x}\right|}_{x=l}=0$$

Applying the conditions of Equation (11) to Equation (12) yields

(13) $$C_{2}=0\;\cos k_{n}l=0$$

Equation (13) shows that, where *G* is the shear elastic coefficient, expressed as $G=\frac{E}{2(1+\nu )}$, and

(14) $$w_{n}=\frac{2n-1}{2l}\pi c_{S}=\frac{2n-1}{2l}\pi \frac{2h}{b}\sqrt{\frac{G}{\rho }}\;n=1,2,3,\ldots$$

Therefore, the natural frequency of the torsional mode can be obtained as follows:

(15) $$f_{n}=\frac{w_{n}}{2\pi }=\frac{\left(2n-1\right)}{2l}\frac{h}{b}\sqrt{\frac{G}{\rho }}\;n=1,2,3,\ldots$$

By substituting the actual geometric dimensions of the cantilever beam into Equation (15), the natural frequency of the torsional mode can be obtained.

Following upon this, Section 3 incorporated Prony series-based viscoelastic parameters into multilayer FEM models to overcome common convergence and shear-locking issues found in nearly incompressible soft materials, offering a practical set of equivalent parameters for dynamic simulation. We established a structured correlation between frequency response, structural design, and material constants, enabling dynamic design-oriented analysis. Therefore, the classical theories presented in Section 2 serve as the theoretical backbone for the novel applications and parameter identification methods developed in this work.

## 3. Experimental Measurement, the Instrument, and Simulation Method

A hybrid additive manufacturing technique was employed, using the PolyJet process (Stratasys J750) based on stereolithography to fabricate composite beam structures composed of alternating soft and hard materials. The soft material was selected based on varying Shore A hardness levels, employing Agilus30 as the viscoelastic property, while the hard material was a general-purpose ABS compatible with the printing system. Since traditional damping composite designs typically incorporate viscoelastic materials sandwiched with a hard layer outside, the composite beam specimens in this study were likewise designed with outer hard surfaces. A steel ball drop test was conducted to impart dynamic impact on the beams, enabling the capture and analysis of structural response signals.

### 3.1. Steel Ball Impact

**Figure 4:**
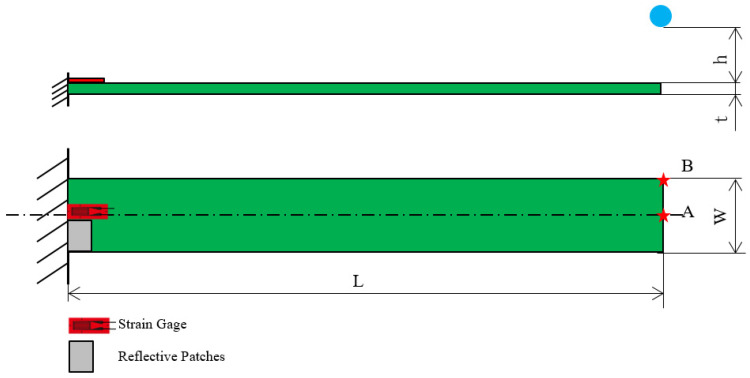
Schematic diagram of cantilever beam.

The fabricated specimens were subjected to dynamic testing under a one-end fixed boundary condition to form a cantilever beam configuration, as illustrated in Figure 4. A stainless steel ball with a diameter of 15.8 mm was released from a controlled height (h) of 64.2 mm through an electromagnet trigger to ensure consistent and repeatable impact at designated locations (Points A and B) along the beam tip. These impact points were carefully selected to induce different modal excitations: Point A primarily triggered bending modes, while Point B excited both bending and torsional responses due to its off-central axis alignment.

The specimens were clamped at one end using a high-rigidity fixture, providing a fixed length of 18 mm and an effective free length of 152 mm (L). The thickness (t) of beams varied based on the material stack design (hard–soft layering). Two synchronized sensing systems were employed to accurately capture the transient structural response: strain gauges adhered to the beam surface near high-strain regions, and a laser Doppler vibrometer (LDV) focused on the beam tip or mid-span to obtain non-contact velocity signals.

**Figure 5:**
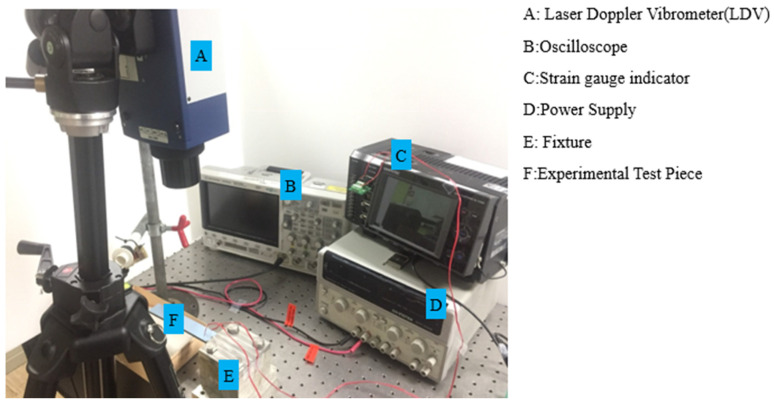
Instrument installation of the ball impact on a beam.

The collected signals were analyzed using Fast Fourier Transform (FFT) to extract dominant frequency components. Additionally, short-time Fourier transform (STFT) was employed to observe the evolution of modal frequencies over time, which is particularly important for identifying transient modes and assessing viscoelastic damping effects. The identified natural frequencies from the first bending mode were used for inverse calculation of material constants, such as Young’s modulus and Poisson’s ratio, based on the Euler–Bernoulli beam theory as outlined in Section 2. These calculated parameters were then used to predict higher-order vibration modes, which were cross-validated with the experimentally measured multi-mode resonant frequencies. The detailed experimental setup, including sensor placements and impact configuration, is illustrated in Figure 5.

### 3.2. Tensile Test

**Figure 6:**
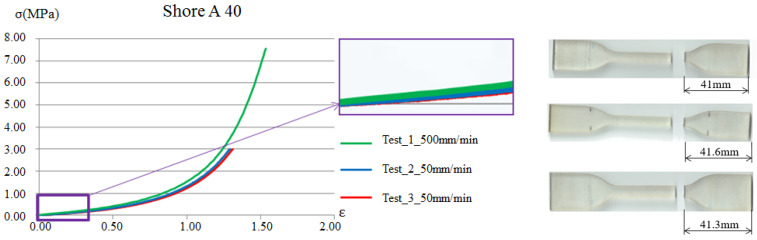
Soft material Shore A 40 tensile test curve.

The tensile test is one of the most representative methods for measuring the mechanical properties of materials. This test involves applying an external force to both ends of a standardized specimen to obtain the relationship between tensile load and elongation, thereby determining the mechanical properties of the material. The test specimens were prepared according to the specified standards, with this experiment following the ASTM-D638 standard. According to the relevant standard, strain rates ranging from 5 to 500 mm/min are acceptable. In this study, tensile tests were conducted at strain rates of 5, 50, and 500 mm/min. Since the Young’s modulus for the viscoelastic material was determined under very small-strain regions, the results presented slight variations across the three strain rates. Therefore, the results shown in Figure 6 correspond to the 50 mm/min strain rate, which demonstrated the best repeatability.

Young’s modulus is typically obtained as the slope of the linear elastic region in the tensile test for general metallic materials. However, in viscoelastic materials, the modulus *E* required for establishing the viscoelastic parameters in the Prony’s series is the instantaneous Young’s modulus *E*_0_, as described in Equation (6). Therefore, by analyzing the force–displacement data obtained from the tensile test, the Young’s modulus is extracted from the initial linear segment of the curve. Figure 6 presents one example of the tensile test results for a soft viscoelastic material with a Shore A hardness value 40. The tensile tests for Shore A hardness 50, 60, 70, 85, and 95 are conducted in the same manner as those for Shore A hardness 40.

### 3.3. Stress Relaxation Test

**Figure 7:**
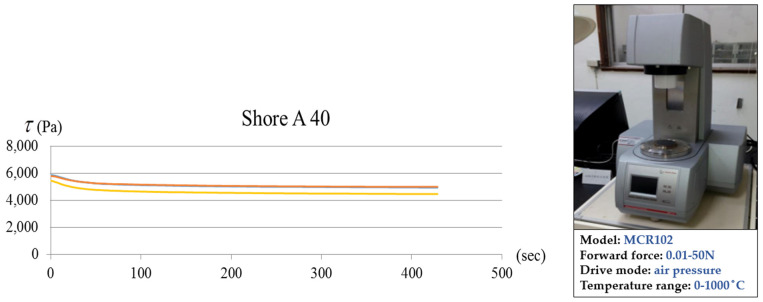
Shore A 40 experimental stress relaxation in the two testing results.

**Figure 8:**
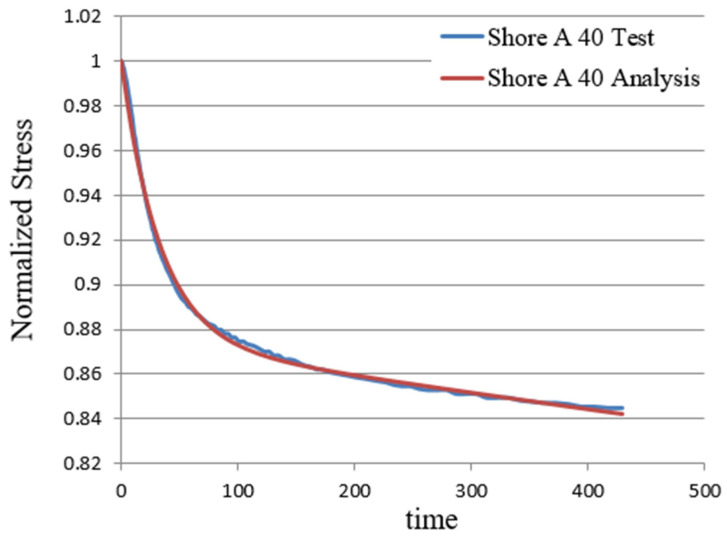
Shore A 40 stress relaxation of soft material compared with simulation and experiment.

The instrument used in this study is a rotational dynamic rheometer, which is designed to analyze the deformation and flow behavior of materials under external forces. Typically, cylindrical specimens are used, and tensile or torsional loading is applied at a controlled temperature. This experiment conducted the test at room temperature (25 °C). Over time, an automatic load reduction mechanism was employed to partially remove the applied load while maintaining a constant total deformation. The reduction in stress over time was recorded to generate a stress relaxation curve. Through this experiment, the relationship between shear stress and time was obtained, as illustrated in Figure 7. The acquired data points were then input into FEM 7.3 software, combined with Young’s modulus obtained from the tensile test, to compute the Prony’s series parameters. Figure 8 compares the experimental stress relaxation results and the numerical analysis, as shown in one example of the soft viscoelastic material with a Shore A hardness value 40. The stress relaxation tests for Shore A hardness 50, 60, 70, 85, and 95 are conducted in the same manner as those for Shore A hardness 40.

### 3.4. Finite Element Simulation

In this study, the commercial finite element software, ABAQUS v.6.13, was employed to perform three-dimensional modeling and dynamic response analysis of composite specimens. All models were constructed based on the actual geometric dimensions of the experimental samples, encompassing various thickness ratios and both symmetric and asymmetric combinations of soft and hard material layers, in order to enhance the accuracy and comparability of the simulation results.

The soft layers were represented using a linear viscoelastic model for material modeling. Material parameters were obtained from stress relaxation experiments and fitted with a Prony series curve. This was subsequently implemented in the viscoelastic module in the time domain to characterize the time-dependent behavior. The hard and soft layers were modeled as isotropic elastic materials on linearly small-strain regions, with Young’s modulus and Poisson’s ratio assigned based on values derived from tensile tests.

To ensure high simulation accuracy and numerical stability, appropriate element types and solvers were selected according to the analysis objectives. For modal analysis of the natural frequencies, 20-node reduced-integration hexahedral elements (C3D20R) were used for meshing, and the Frequency module in ABAQUS was applied to solve for the natural frequencies and corresponding mode shapes. For dynamic impact simulations, 8-node reduced-integration hexahedral elements (C3D8R) were employed with an explicit solver. The excitation was applied as a concentrated force pulse at the same location as in the experiments, while the boundary condition was set as a cantilever beam with one end fixed, consistent with the experimental setup.

The simulation results included the extraction of modal frequencies of the structure, which were validated against experimental data obtained from laser Doppler vibrometry (LDV). It is important to note that, based on the discussion in Section 4, the Young’s modulus values assigned in the finite element method for both soft and rigid materials were input through inverse calculation from the resonant frequencies of the overall structure under steel ball impact, rather than using individually measured values obtained from tensile tests. This approach was adopted to avoid inaccuracies arising from mesh locking and convergence issues during simulation, and to enable more accurate prediction of the dynamic response of the composite structure. It is an essential concept emphasized in this paper.

## 4. Results and Discussion

To investigate the influence of material combinations on structural vibration behavior, 11 configurations of cantilever beam specimens were fabricated and subjected to dynamic impact tests. Five pieces of standard specimens for a specific viscoelastic material were prepared for testing in a rotational dynamic rheometer. For the steel ball impact tests, three specimens were fabricated for each type of composite sample, and each specimen underwent five repeated tests. The experiments confirmed repeatability, and the results were compiled and presented in this study. Each specimen had a length of 170 mm, a width of 25 mm, and a total thickness of 3 mm. The soft material layers varied in Shore A hardness (A40, A50, A60, A70, A85, A95) and thickness, and were arranged in both symmetric and asymmetric stacking sequences. Additionally, this study further investigates the effect of increasing the thickness of soft materials (0.9 mm, 1.2 mm, 1.5 mm, and the original in 0.6 mm) to enhance viscoelastic properties on the dynamic characteristics relevant to structural stiffness of the composite structure. All specimens were fabricated using stereolithography additive manufacturing to ensure material continuity and geometric precision, and they were tested under consistent conditions for both impact excitation and modal analysis.

**Figure 9:**
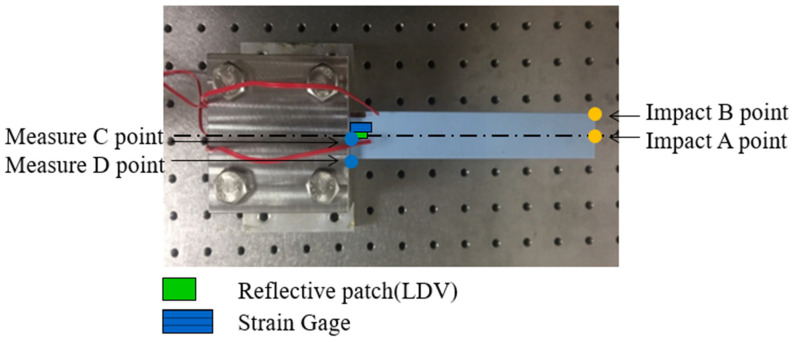
Dynamic simulation impact point and measurement point.

Dynamic impact experiments and finite element simulations (FEM) were conducted concurrently, with impact locations illustrated in Figure 9. The results showed that when impacted at point A, only bending modes were excited and observed at measurement points C and D. In contrast, impact at point B enabled both bending and torsional modes to be captured at point D. Based on modal identification, it was determined that only the configuration with impact at point B and measurement at point D could effectively excite and observe both bending and torsional modes. Therefore, this configuration was adopted as the standard condition for all subsequent experimental and simulation analyses. Moreover, the single-mode response observed at point C under impact at point A was used as a reference to isolate and estimate torsional modes, thereby assisting in mode separation and identification. The selected specimen configurations and parameters in this study effectively demonstrate the critical influence of material combinations on structural vibration characteristics.

### 4.1. Symmetrical Model with the Same Thickness Ratio

**Figure 10:**
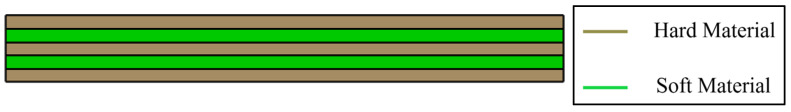
Schematic diagram of a thickness section compounded by hard material (brown) and soft material (green).

A fundamental symmetric model was initially established to investigate the dynamic characteristics of multilayered composite structures, followed by the development of both experimental and analytical models. The composite specimen consisted of five layers, alternating between a rigid material and a soft material, including Shore A hardness 40, 50, 60, 70, 85, 95. Each layer had a thickness of 0.6 mm, with top, middle, and bottom layers composed of rigid material, while the middle layers embedded in between consisted of soft material, as shown in Figure 10.

**Figure 11:**
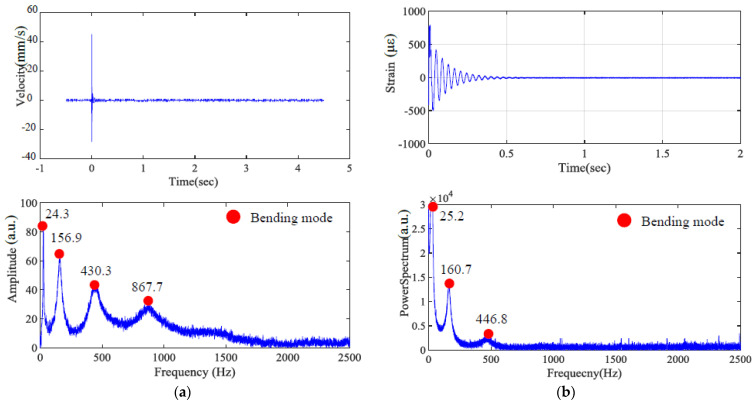
Time–frequency domain of hard material and soft Shore A 40 (Impact point A); (**a**) LDV, (**b**) strain gauge.

**Figure 12:**
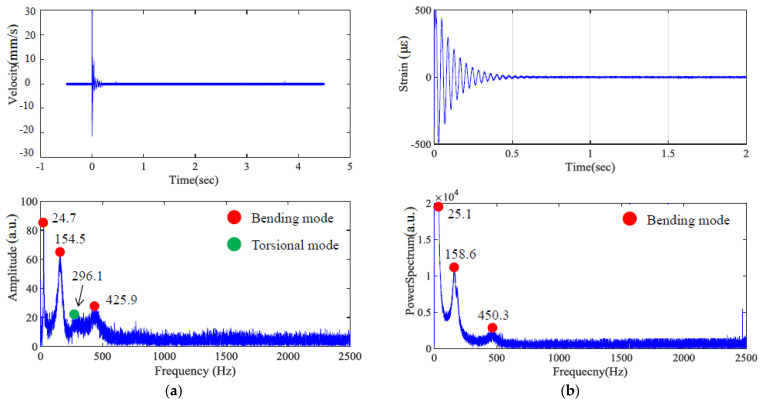
Time–frequency domain of hard material and soft Shore A 40 (Impact point B) by (**a**) LDV and (**b**) strain gauge, respectively.

**Figure 13:**
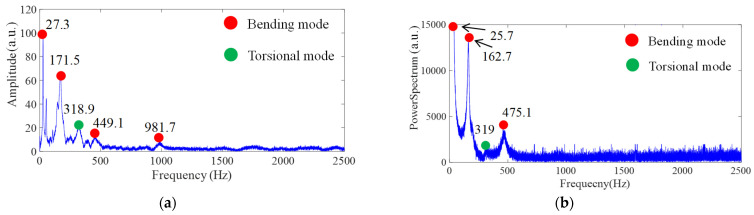
Frequency spectrum of hard material and soft Shore A 95 (Impact point B) by (**a**) LDV and (**b**) strain gauge, respectively.

The specific layer is assumed to be homogeneous and isotropic. The originally designed dimensions were 170 × 25 × 3 mm; however, due to manufacturing process factors, the final dimensions were measured as 170 × 25.16 × 3.08 mm. The experimental boundary condition was a cantilever beam with a fixed end, where 18 mm of the specimen was clamped, reducing the effective length to 152 mm. Although this study primarily investigates bending modes, i.e., the most easily excited vibration type, torsional modes are also dedicated to exciting for identifying the dynamic property of the dual material. Figure 11, Figure 12 and Figure 13 present the impact of transient response and transferring as a frequency spectrum by fast Fourier transformation. As illustrated in Figure 11 and Figure 12, the hard material combined with soft Shore A 40 demonstrates the impact results at points A and B, respectively, based on measurements taken using both LDV and a strain gauge. It can be observed that only impacting point B is capable of exciting the torsional mode marked as a green dot. However, with the softer Shore A 40 material, the excitation of the torsional mode is less prominent. As shown in Figure 13, when the soft material is replaced with a slightly harder Shore A 95, the excitation and identification of the torsional mode become significantly easier.

Table 1 and Table 2 present the FEM, experimental, and theoretical results in natural frequencies. A comparative analysis was conducted between cases with and without the inclusion of viscoelastic coefficients. Table 3 summarizes the material parameters obtained from experimental tests and measurements for all symmetric models. In the initial definition of the dual-material parameters, separate experiments were conducted for the hard and soft materials to determine their material properties individually. Subsequently, both materials were combined, and the resonant frequency was validated using the steel ball impact method. However, during the finite element method, the convergence issue and shear-locking phenomenon arose due to Poisson’s ratio of the viscoelastic material approaching 0.5 and the dramatic difference in rigidity between soft and hard materials. To address this, the Young’s modulus used in the analysis was derived from the equivalent stiffness of the dual-material laminated model subjected to vibration. In other words, regardless of theoretical analysis or FEM, Poisson’s ratio is represented as the equivalent material constant calculated as a bulk structure based on the impact-induced vibration of a cantilever beam to serve as the input value for analysis.

Table 1 and Table 2 compare various results based on different FEM models, using experimental measurements at impact points A and B as referred benchmarks for comparison. In the first column of the FEM result, based on the viscoelastic material model, it is anticipated that implicit analysis with the viscoelastic material parameter model using the analytical method of natural frequency will be the most accurate and efficient approach. Comparisons include implicit analysis without viscoelastic material parameters for natural frequency, i.e., second column, explicit analysis with viscoelastic material parameters at impact point C (capable of exciting only bending modes, third column) and impact point D (capable of exciting both bending and torsional modes, fourth column) through the time–frequency transformation of transient responses. The results are further compared with the natural frequency, the values of bending and torsional models of the Euler–Bernoulli beam in homogeneous and isotropic material without viscoelastic characteristics.

From Table 1, the bending resonant frequency from the ball-drop impact measured at point A by LDV measurement, compared with the natural frequencies of FEM considering viscoelastic material properties, shows a maximum error of 2.72% in the sixth mode. The maximum error using strain gauge measurements occurs in the fifth mode, with an error of 3.33%. The test results of impact at point B in Table 2 present that the maximum error in bending mode resonant frequency measured by LDV is 1.5% in the fifth mode, while the maximum error using strain gauge measurements is also in the fifth mode, at 5.28%. This demonstrates that FEM simulation results when considering viscoelastic material parameters, provide accurate predictions compared to experimental measurements. Conversely, if FEM simulation does not consider viscoelastic material constants, almost every mode shows significant deviations, with errors ranging from about 7% to 12%.

Discussion of FEM calculations compared to the beam theory for isotropic and homogeneous materials shows that natural frequencies of theoretical analysis, assuming an equivalent bulk material for the structure without viscoelastic properties in the second and fourth soft layers, results in larger errors for some high-frequency modes, particularly mixed modes, and torsional modes.

If we discuss FEM-explicit calculations for impacts at points A and B, extracting results at points C and D, they correspond with experimental measurements. That is, using a central off-axis position for ball-drop impact and sensing points outside the central axis of the structure allows for the simultaneous bending and torsional mode effects. In the transient response of the FEM-explicit model, using material constants of equivalent bulk material, including viscoelastic material properties, accurately correlates the natural frequencies of FEM-implicit model results and experimental measurements.

**Figure 14:**
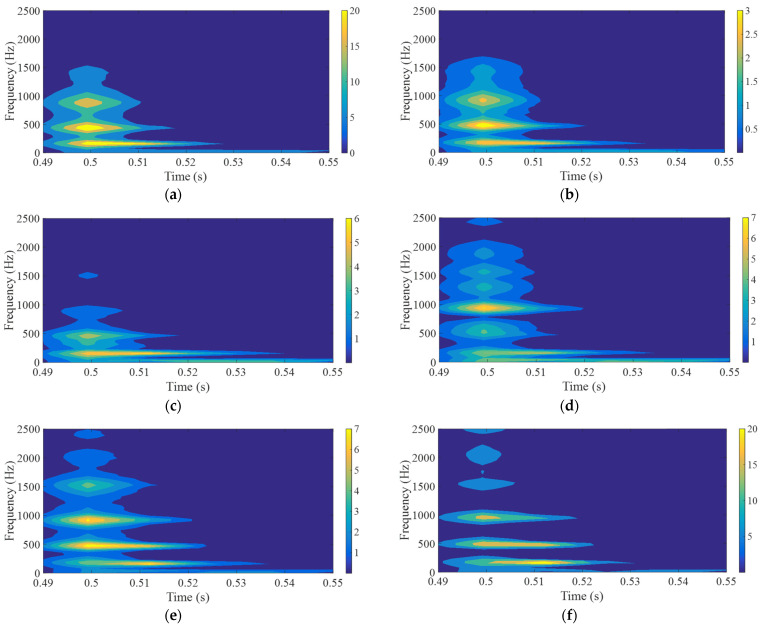
Short-time Fourier transforms of hard–soft dual material (Impact point A), (**a**) Shore A 40, (**b**) 50, (**c**) 60, (**d**) 75, (**e**) 85, (**f**) 95.

Finally, using transient response to conduct a short-time Fourier transform (STFT) of the ball impact on a composite structure made of hard and soft materials, the frequency attenuation of symmetric structures with different Shore hardness A values in the second and fourth layers can be observed, as shown in Figure 14. Softer material combinations, e.g., Shore A hardness, experience faster attenuation to show shorter time duration at low frequencies. Continuous energy intensities across the different frequency ranges indicate a wider resonant frequency bandwidth. Conversely, with material combinations transitioning from soft to hard (Shore A hardness 40 to 95), harder material combinations, i.e., Shore A hardness 95, exhibit slower attenuation to longer duration at low frequencies. Less continuous energy intensities across the different frequency ranges indicate a narrower resonant frequency bandwidth.

### 4.2. Asymmetric Model with the Same Thickness Ratio

**Figure 15:**
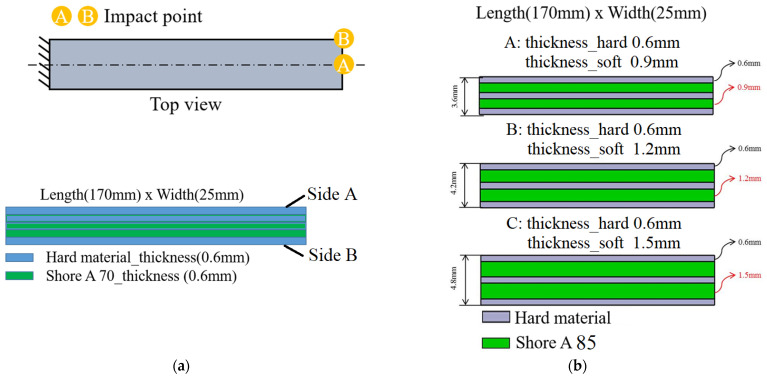
(**a**) Asymmetric multilayer structures are composed of heterogeneous materials, and (**b**) symmetric multilayer structures have varied in the proportion of soft and rigid materials.

Based on the previous modal analysis of symmetric specimens, it was confirmed that the dynamic characteristics of viscoelastic materials can be observed across both high- and low-frequency ranges. This validates the feasibility of stereolithographic fabrication methods for producing multilayer composite structures. This section investigates an asymmetric laminated rectangular specimen under identical material composition conditions to evaluate its dynamic behavior. The fabricated specimen comprises six layers with dimensions of 170 × 25 × 3 mm and an individual layer thickness of 0.6 mm. Layers 1, 4, and 5 are rigid, while layers 2 and 3 are viscoelastic soft material. The soft material employed in this study corresponds to Shore A 70. This section focuses on adjustments to the proportion of soft and rigid materials, discussing several structures in which the thickness of the soft material gradually increases to verify whether the previously discussed analytical methods remain applicable. The asymmetric structures in the subsection and the variations in the proportion of soft and rigid materials in Section 4.3 are represented by the test structures shown in Figure 15a and Figure 15b, respectively.

This study takes the FEM incorporating viscoelastic material behavior based on the Prony series as the reference for evaluating the accuracy of experimental measurements. The differences in ball drop impact positions on Sides A and B, as indicated in Figure 15a, were analyzed to verify whether impacts on structures with less soft material on Side A versus more soft material on Side B would influence the measurement results. To simplify the explanation from the previous section regarding the drop impact positions, only experimental results that simultaneously excite both bending and torsional modes are presented below. The corresponding natural frequencies for each mode are summarized in Table 4, including comparisons between FEM results from those with and without viscoelastic material parameters.

The maximum deviation of the bending mode natural frequency listed in Table 4, obtained from FEM with viscoelastic material parameters, compared with the frequency experimentally measured by LDV, was the eighth mode with an error of 5.42%, while the frequency measured by strain gauge occurred at the first mode with an error of 5.65%. The torsional natural frequency obtained from FEM with viscoelastic material parameters, compared with that experimentally measured by LDV, was an error of 5.04%. From the perspective of experimental measurements, the results of impacts on Sides A and B show that the experimental results from Side B have a slightly larger deviation compared to the FEM results. This may be because Side B is closer to a distribution of softer materials, leading to an actual attenuation effect in transmitting vibrations. In contrast, FEM only represents the resonance frequency characteristics of the overall structure. In the FEM model, assuming an equivalent homogeneous structure is considered with and without the viscoelastic effects, the modal analysis shows a maximum deviation of 3.46% at the eleventh bending mode, 11.67% at the mixed mode, and 5.12% at the third torsional mode (9th mode). The error tends to increase with higher and more complicated vibration modes. However, the bending mode discussion, in comparison with theoretical results, reveals a maximum error of 0.82% at the first mode and a minimum of 0.02% at the eighth mode, demonstrating a trend of decreasing error with increasing mode number when Prony parameters in FEM are included in the third and fourth layers. This result again confirms the importance of the dynamic behavior of viscoelastic materials at lower frequency regions.

From the calibration perspective of material property, Young’s modulus was estimated as 1.73 GPa, based on the LDV measurement of the first bending mode under impact on Side A. Comparing the discussion in Section 4.1 and the material combinations shown in Table 3, which consist of the same proportion of hard and soft materials (Shore A 70), the symmetric (1.58 GPa) and asymmetric (1.73 GPa) material stacking configurations significantly influence the dynamic response and have an essential impact. In other words, both experiments also demonstrate that even with identical total thickness and layer proportion of hard and soft materials, variation in the stacking sequence alone can lead to either an increase or decrease in the stiffness of the structure. This demonstrates the critical reference value of this study for the design of multilayer composite structures.

Due to the high porosity of 3D-printed materials, formerly regarded as one of the most accurate approaches, the intrinsic material constants of inverse calculation by the conventional ultrasonic measurement method [26] cannot be effectively applied. Similar to the vibration-based parameter identification used in this study, our research has succeeded in determining complex multiphysical material constants for piezoceramic materials using vibration characteristics combined with genetic algorithms, with the results validated through both experiments and simulations in a previous study [27]. When compared with material constants obtained from traditional tensile testing or emerging multiscale mechanical computations for 3D-printed materials, the vibration-based approach has demonstrated consistent trends and reliable correspondence [28]. Nevertheless, it is inevitable that since vibration excitation constitutes a perturbation signal, the dynamic response reflects only linear, elastic deformations in the initial portion of the stress–strain curve. As a result, the Young’s modulus estimated from the slope may be slightly overestimated.

### 4.3. Symmetry Model with Different Thickness Ratios

To further investigate the influence of soft viscoelastic layer thickness on torsional mode response, this study conducted dynamic impact simulations and experimental testing using three different thickness configurations. Excitation was applied at point B, and responses were measured at point D to effectively excite both bending and torsional modes of the structure. All specimens were designed as symmetric five-layer laminates, with rigid layers of 0.6, 0.9, and 1.2 mm thickness and soft viscoelastic layers made of Shore A 85 elastomer, as shown in Figure 15b.

As summarized in Table 5 and Table 6, FEM simulations incorporating Prony series viscoelastic parameters applied to layers 2 and 4 were analyzed for torsional mode response error trends relative to thickness variations. For the 0.9 mm specimens, the maximum error in natural frequencies of the torsional modes obtained from FEM simulations compared to LDV experimental measurements was 9.21% at the fourth mode. In contrast, strain gauge measurements showed a maximum error of 10.49%. At 1.2 mm thickness, the maximum error in the fourth mode increased to 11.49%; for 1.5 mm, before reaching 11.94%. The overall results show a decreasing error trend with increasing mode order, and thinner viscoelastic layers suppress variation in high-order torsional modes more effectively. The thickness of the soft materials has slight influence on the resonant frequency in both FEM and experimental results for bending modes, with errors in the first four bending modes all within 5.51%.

As listed in Table 7, the maximum errors for bending and torsional modes observed in the dynamic experiments across all configurations did not exceed 5.51% and 11.94%, respectively, aligning well with the FEM simulations incorporating viscoelastic parameters. Conversely, compared to FEM models that neglect viscoelastic behavior, that is, treating the structure as an equivalent homogeneous material, the torsional mode frequency errors reached up to 16.03% across the three thickness configurations. This highlights the severe underestimation of frequency response differences when the actual energy dissipation behavior of materials is excluded. This confirms the effectiveness of the Prony series-based modeling approach in predicting torsional dynamic responses and underscores the strong correlation between soft-layer thickness and energy dissipation mechanisms.

The equivalent Young’s modulus estimated from LDV measurements was approximately 1.25 GPa, further validating the appropriateness of the FEM material parameter settings and supporting the use of Prony series viscoelastic modeling for accurately predicting torsional dynamic behavior.

## 5. Conclusions

For the hybrid structure of soft and hard materials, the parameter analysis of mechanical properties and the accuracy of dynamic properties have the following key conclusions:

Stiffness tests for structure combined with experiments in rheometer-based stress relaxation can quickly and accurately determine Prony series viscoelastic parameters to establish viscoelastic material parameters. When applied to layered structures composed of soft and hard materials, accurate dynamic characteristics can be obtained using the finite element method. Based on a Shore A hardness of 40–95 for soft materials and a distribution where hard materials account for more than 50% of the volume, using the equivalent Young’s modulus of the overall structure along with the Prony series viscoelastic parameters eliminates the need for the Young’s modulus of viscoelastic materials. This method significantly saves the time and costs required for parameter estimation. The FEM and experimental results exhibit strong consistency. Furthermore, the structure demonstrates the frequency-dependent stiffness characteristics of viscoelastic materials, where it is observed that at low frequencies, the structure exhibits weaker stiffness but stronger vibration absorption capabilities. In contrast, stiffness increases at high frequencies while vibration absorption decreases.

However, for asymmetric soft–hard material hybrid structures and the cases where the exceeded half proportion of soft materials increases, dynamic measurements conducted on the structural surface with more soft material distribution reveal significant discrepancies on resonant frequency compared with the FEM results. As the thickness of the soft material increases, the discrepancies also tend to increase. One reason is attributed to the limitations of Euler–Bernoulli beam theory, as the increasing thickness of soft materials leads to a decrease in the length-to-thickness and width-to-thickness ratios, causing error amplification. Nevertheless, for symmetric multilayered structures with a soft–hard material thickness ratio of approximately exceed 2.5:1, experimental results remain highly correlated with FEM simulations based on the Prony series, with deviations still within acceptable limits.

Therefore, as layered additive manufacturing increasingly enables the production of functional vibration–reduction structures composed of soft–hard hybrid material, this study proposes an FEM model based on viscoelastic Prony series but utilizing the equivalent Young’s modulus of the structure, proving to be an effective and reliable method for predicting dynamic characteristics.

## Figures and Tables

**Table 1:** Natural frequency (Unit in Hz) of hard material with soft shore A 40 impact point A. Symbols after the bottom line under Mode are indicated as B (bending), T (torsional), or no mark (mixed mode). The errors are in brackets.

Mode	FEM—Implicit		FEM—Explicit		Experiment		Theory
	Natural Frequency		FFT—Transient Response				
	w/Prony	w/o Visco	w/Prony (pt. C)	w/Prony (pt. D)	LDV	Strain Gauge	
1_B	24.7	22.9	25.5	25.5	24.3	25.2	24.3
		−(7.29)	(3.24)	(3.24)	−(1.62)	(2.02)	−(1.62)
2_B	154.8	143.2	153.6	153.6	156.9	160.7	152.2
		−(7.49)	−(0.78)	−(0.78)	(1.36)	(3.81)	−(1.68)
5_B	432.4	400.8	428.4	428.4	430.3	446.8	426.5
		−(7.31)	−(0.93)	−(0.93)	−(0.49)	(3.33)	−(1.36)
6_B	844.7	784.2	837.6	837.6	867.7		835.5
		−(7.16)	−(0.84)	−(0.84)	(2.72)		−(1.09)
8_B	1391.2	1292	1380	1380			1381.4
		−(7.13)	−(0.81)	−(0.81)			−(0.70)
11_B	2069.8	1928	2055	2055			2063.5
		−(6.85)	−(0.72)	−(0.72)			−(0.30)
3	223.6	197.8					198.2
		−(11.54)					−(11.36)
10	1273.4	1120.8					1242.3
		−(11.98)					−(2.44)
4_T	317.8	289.2			296.1		296.1
		−(9.00)			−(6.83)		−(6.83)
7_T	964	878.2					889.2
		−(8.90)					−(7.76)
9_T	1641.9	1498.2					1482.1
		−(8.75)					−(9.73)

**Table 2:** Natural frequency (Unit in Hz) of hard material with soft shore A 40, impact point B. Symbols after the bottom line under Mode are indicated as B (bending), T (torsional), or no mark (mixed mode). The errors are in brackets.

Mode	FEM—Implicit		FEM—Explicit		Experiment		Theory
	Natural Frequency		FFT—Transient Response				
	w/Prony	w/o Visco	w/Prony (pt. C)	w/Prony (pt. D)	LDV	Strain Gauge	
1_B	24.7	22.9	25.4	25.4	24.7	25.1	24.3
		−(7.29)	(2.83)	(3.24)	(0.00)	−(1.18)	−(1.62)
2_B	154.8	143.2	153.3	153.3	154.5	158.6	152.2
		−(7.49)	−(0.97)	−(0.78)	−(0.19)	(3.81)	−(1.68)
5_B	432.4	400.8	427.7	427.7	425.9	450.3	426.5
		−(7.31)	−(1.09)	−(0.93)	−(1.50)	(5.28)	−(1.36)
6_B	844.7	784.2	836.1	836.1			835.5
		−(7.16)	−(1.02)	−(0.84)			−(1.09)
8_B	1391.2	1292	1379	1379			1381.4
		−(7.13)	−(0.88)	−(0.81)			−(0.70)
11_B	2069.8	1928	2053	2053			2063.5
		−(6.85)	−(0.81)	−(0.72)			−(0.30)
3	223.6	197.8					198.2
		−(11.54)					−(11.36)
10	1273.4	1120.8					1242.3
		−(11.98)					−(2.44)
4_T	317.8	289.2		312.2	296.1		296.1
		−(9.00)		−(1.76)	−(6.83)		−(6.83)
7_T	964	878.2		944.1			889.2
		−(8.90)		−(2.06)			−(7.76)
9_T	1641.9	1498.2		1605			1482.1
		−(8.75)		−(2.25)			−(9.73)

**Table 4:** Natural frequency (Unit in Hz) of hard material with soft Shore A 70. Symbols after the bottom line under Mode are indicated as B (bending), T (torsional), or no mark (mixed mode). The errors are in brackets.

Mode	FEM—Implicit		FEM—Explicit		Experiment		Experiment		Theory
	Natural Frequency				LDV		Strain Gauge		
	w/Prony	w/o Visco	Side A	Side B	Side A	Side B	Side A	Side B	
1_B	26.92	26.05	27.93	27.9	26.9	26.5	25.6	25.4	26.7
		−(0.03)	(3.75)	(3.64)	(0.82)	−(1.56)	−(4.9)	−(5.65)	−(0.82)
2_B	168.45	162.95	168.2	168.4	172.1	170.3	166.4	165.7	167.3
		−(0.03)	−(0.15)	−(0.03)	(2.17)	(2.52)	−(1.63)	−(1.63)	−(0.68)
5_B	470.87	455.32	466.7	469	488.9	486.7	477.9	478.6	468.65
		−(3.3)	−(0.89)	−(0.4)	(3.83)	(3.15)	(1.49)	(5.28)	−(0.47)
6_B	920.62	889.81	917.1	918.8	955.3				918.11
		−(3.35)	−(0.38)	−(0.2)	(3.77)				−(0.27)
8_B	1517.7	1466.1	1513	1517	1600				1517.93
		−(3.4)	−(0.31)	−(0.05)	(5.42)				(0.02)
11_B	2260	2181.9	2259	2261					2267.36
		−(3.46)	−(0.04)	−(0.05)					(0.33)
3	241.01	214.17							223.92
		−(11.13)							−(7.09)
10	1374.1	1213.8							1403.1
		−(11.67)							(2.11)
4_T	331.47	314.75	330.9	331.1	315.9	323.3			315.9
		−(5.04)	−(0.17)	−(0.03)	(5.04)	−(2.46)			−(4.7)
7_T	1005.3	955.28	998.8	1002					947.7
		−(4.98)	−(0.65)	−(0.33)					−(5.73)
9_T	1713.1	1628.6	1704	1707					1579.5
		−(5.12)	−(0.53)	−(0.36)					−(7.8)

**Table 5:** Natural frequency (Unit in Hz) of hard material with soft Shore A 85 (0.9 mm thickness) impact point B. Symbols after the bottom line under Mode are indicated as B (bending), T (torsional), or no mark (mixed mode). The errors are in brackets.

Mode	FEM—Implicit		FEM—Explicit	Experiment		Theory
	Natural Frequency					
	w/ Prony	w/o Visco	(pt. D)	LDV	Strain Gauge	
1_B	29.4	27.1	29.1	28.3	28.5	28.9
		−(7.82)	−(1.02)	−(3.74)	−(3.06)	−(1.7)
2_B	184.2	169.8	181.9	184.1	183.2	181.1
		−(7.82)	−(1.25)	−(0.05)	−(0.54)	−(1.68)
5_B	514.4	473.8	509.6	508.3	529.8	507.3
		−(7.89)	−(0.93)	−(1.19)	(2.99)	−(1.38)
6_B	1003.7	924.1	996.2	1059		993.8
		−(7.93)	−(0.75)	(5.51)		−(0.99)
8_B	1650.6	1519	1639			1643
		−(7.97)	−(0.7)			−(0.46)
11_B	2451.8	2255	2451			2454.2
		−(8.03)	−(0.03)			(0.1)
3	212.4	186.4				200
		−(12.24)				−(5.84)
10	1222.5	1065				1253.2
		−(12.88)				(2.51)
4_T	392.9	345.1	385.5	356.7	351.7	356.7
		−(12.17)	−(1.88)	−(9.21)	−(10.49)	−(9.21)
7_T	1188.4	1045.3	1168			1059
		−(12.04)	−(1.72)			−(10.89)
9_T	2014.7	1775.6	1976			1783.5
		−(11.87)	−(1.92)			−(11.48)

**Table 6:** Natural frequency (Unit in Hz) of hard material with soft Shore A 85 (1.2 mm thickness) at impact point B. Symbols after the bottom line under Mode are indicated as B (bending), T (torsional), or no mark (mixed mode). The errors are in brackets.

Mode	FEM—Implicit		FEM—Explicit	Experiment		Theory
	Natural Frequency					
	w/Prony	w/o Visco	(pt. D)	LDV	Strain Gauge	
1_B	35	31.6	35.2	33.7	32.1	33.3
		−(9.71)	(0.57)	−(3.71)	−(8.29)	−(4.86)
2_B	218.7	197.5	215.5	218.3	204.9	208.6
		−(9.69)	−(1.46)	−(0.18)	−(6.31)	−(4.62)
5_B	610	550.6	599.4	622.5		584.5
		−(9.74)	−(1.74)	(2.05)		−(4.18)
6_B	1188.4	1072.1	1168			1145.1
		−(9.79)	−(1.72)			−(3.64)
8_B	1951.3	1758.6	1917			1893.2
		−(9.88)	−(1.76)			−(2.98)
11_B	2893.5	2604.6	2836			2827.8
		−(9.98)	−(1.99)			−(2.27)
3	216.4	185.9				199.5
		−(14.09)				−(7.81)
10	1242.1	1059				1249.9
		−(14.74)				(0.63)
4_T	451.4	387.1	441.6	400.9		400.9
		−(14.24)	−(2.17)	−(11.19)		−(11.19)
7_T	1367	1173	1336			1202.7
		−(14.19)	−(2.27)			−(12.02)
9_T	2320.3	1994.9	2259			2004.5
		−(14.02)	−(2.64)			−(0.14)

**Table 7:** Natural frequency (Unit in Hz) of hard material with soft Shore A 85 (1.5 mm thickness) at impact point B. Symbols after the bottom line under Mode are indicated as B (bending), T (torsional), or no mark (mixed mode). The errors are in brackets.

Mode	FEM—Implicit		FEM—Explicit	Experiment		Theory
	Natural Frequency					
	w/Prony	w/o Visco	(pt. D)	LDV	Strain Gauge	
1_B	39.4	34.8	37.9	36.7	33.4	36.7
		−(11.68)	−(3.81)	−(6.85)	−(15.23)	−(6.85)
2_B	246.2	217.7	236.8	240.9	220.2	229.9
		−(11.58)	−(3.82)	−(2.15)	−(10.56)	−(6.62)
5_B	685.7	606.1	658.4	36.7		644.2
		−(11.61)	−(3.98)	−(6.85)		−(6.05)
6_B	1332.1	1177.9	1280			1261.9
		−(11.58)	−(3.91)			−(5.23)
8_B	2180.8	1927.9	2094			2086.4
		−(11.6)	−(3.98)			−(4.33)
11_B	3223.1	2848.1	3087			3116.5
		−(11.63)	−(4.22)			−(3.31)
3	213.2	179.3				192.3
		−(15.9)				−(9.8)
10	1220.1	1017.8				1205.4
		−(16.58)				(2.11)
4_T	489.1	410.7	466.9	430.7		430.7
		−(16.03)	−(4.54)	−(11.94)		−(1.2)
7_T	1480.6	1245.1	1411			1292.1
		−(15.9)	−(4.7)			−(12.73)
9_T	2512.5	2117.9	2282			2153.5
		−(15.71)	−(9.17)			−(14.29)

**Table 3:** Material property of the hard and soft hybrid material.

		Hard Material Combination Soft Shore A 40	Hard Material Combination Soft Shore A 50	Hard Material Combination Soft Shore A 60	Hard Material Combination Soft Shore A 70	Hard Material Combination Soft Shore A 85	Hard Material Combination Soft Shore A 95
Young’s modulus (GPa)		1.33	1.51	1.56	1.58	1.6	1.7
Poisson’s ratio		0.116	0.196	0.09	0.144	0.161	0.211
Viscoelastic coefficient in Prony series	g_1_	0.125	0.118	0.131	0.11	0.23	0.156
	g_2_	0.374	0.381	0.365	0.389	0.735	0.656
	k_1_	0	0	0	0	0	0
	k_2_	0	0	0	0	0	0
	τ_1_	32.99	40.3	41.6	58.57	7.45	8.66
	τ_2_	4717	9773	5746	7754	17.62	34.05

## Data Availability

The data reported results can be found in the paper, and also connect to the corresponding author.
